# GreenPhylDB v5: a comparative pangenomic database for plant genomes

**DOI:** 10.1093/nar/gkaa1068

**Published:** 2020-11-25

**Authors:** Guignon Valentin, Toure Abdel, Droc Gaëtan, Dufayard Jean-François, Conte Matthieu, Rouard Mathieu

**Affiliations:** Bioversity International, Parc Scientifique Agropolis II, 34397 Montpellier, France; French Institute of Bioinformatics (IFB)—South Green Bioinformatics Platform, Bioversity, CIRAD, INRAE, IRD, F-34398 Montpellier France; Syngenta Seeds SAS, 31790 Saint-Sauveur France; French Institute of Bioinformatics (IFB)—South Green Bioinformatics Platform, Bioversity, CIRAD, INRAE, IRD, F-34398 Montpellier France; AGAP, Univ de Montpellier, CIRAD, INRAE, Montpellier SupAgro, F-34398 Montpellier, France; CIRAD, UMR AGAP, F-34398 Montpellier, France; French Institute of Bioinformatics (IFB)—South Green Bioinformatics Platform, Bioversity, CIRAD, INRAE, IRD, F-34398 Montpellier France; AGAP, Univ de Montpellier, CIRAD, INRAE, Montpellier SupAgro, F-34398 Montpellier, France; CIRAD, UMR AGAP, F-34398 Montpellier, France; Syngenta Seeds SAS, 31790 Saint-Sauveur France; Bioversity International, Parc Scientifique Agropolis II, 34397 Montpellier, France; French Institute of Bioinformatics (IFB)—South Green Bioinformatics Platform, Bioversity, CIRAD, INRAE, IRD, F-34398 Montpellier France

## Abstract

Comparative genomics is the analysis of genomic relationships among different species and serves as a significant base for evolutionary and functional genomic studies. GreenPhylDB (https://www.greenphyl.org) is a database designed to facilitate the exploration of gene families and homologous relationships among plant genomes, including staple crops critically important for global food security. GreenPhylDB is available since 2007, after the release of the *Arabidopsis thaliana* and *Oryza sativa* genomes and has undergone multiple releases. With the number of plant genomes currently available, it becomes challenging to select a single reference for comparative genomics studies but there is still a lack of databases taking advantage several genomes by species for orthology detection. GreenPhylDBv5 introduces the concept of comparative pangenomics by harnessing multiple genome sequences by species. We created 19 pangenes and processed them with other species still relying on one genome. In total, 46 plant species were considered to build gene families and predict their homologous relationships through phylogenetic-based analyses. In addition, since the previous publication, we rejuvenated the website and included a new set of original tools including protein-domain combination, tree topologies searches and a section for users to store their own results in order to support community curation efforts.

## INTRODUCTION

Plant comparative genomics resources usually compare reference genomes to compute homology sequences and enable functional annotation transfer (1,2,3,4,5). However, with the growing number of whole genome sequences available within the same species, it has been shown than a single reference is not enough to capture its total genetic diversity (6). A pangenome, usually defined as the full gene repertoire within a species, can be partitioned into core genes that are shared by all individuals and dispensable genes that are present only in a subset of individuals (6,7,8). Characterizing them can have a great potential in plants for crop improvement (7,9,10) as candidate genes can potentially be missing in the genotype used to set up a reference genome. Pangenomic studies have recently been conducted in several crops, revealing significant differences with presence absence variations (PAVs) and/or copy number variations across genotypes (11,12,13,14,15,16,17,18). It became obvious that distinguishing core from dispensable genes is important as dispensable genes can be associated with useful trait diversity (10). Finally, PAVs can have an influence on orthology detection as specific genotype gene losses can lead to false negative results in interspecific comparisons or to pseudo-orthology (19).

Until now, comparative genomics databases have not fully taken advantage of these new datasets. Here, we present an updated version of the GreenPhylDB, a database that features multiple genomes for 19 species (e.g. rice, maize, banana, grape and cacao) as well as 27 other species with single reference genomes, for a total of 46 genomes. Publicly available genomes were processed to generate representative pangenes (i.e. a set of representative or consensus sequences) for species that were used in multi-species sequence clustering. Resulting gene families were functionally annotated and analysed with orthology detection methods.

## DATABASE CONSTRUCTION

### Sequence retrieval and quality checks

We retrieved 132 publicly available datasets (coding DNA sequence and protein-coding genes) for 46 (Supplementary Table S1) and assessed their gene annotation predictions using BUSCO Plants v3.0.2 (embryophita_odb10) (20). We checked that the number of CDS was consistent with the number of proteins and that they shared the same locus tag name. When protein-coding genes were missing, we generated the sequences from GFF files originating from data providers. Finally, alternate splices were filtered, and the longest sequence was conserved.

### Pangene construction

**Figure 1.**
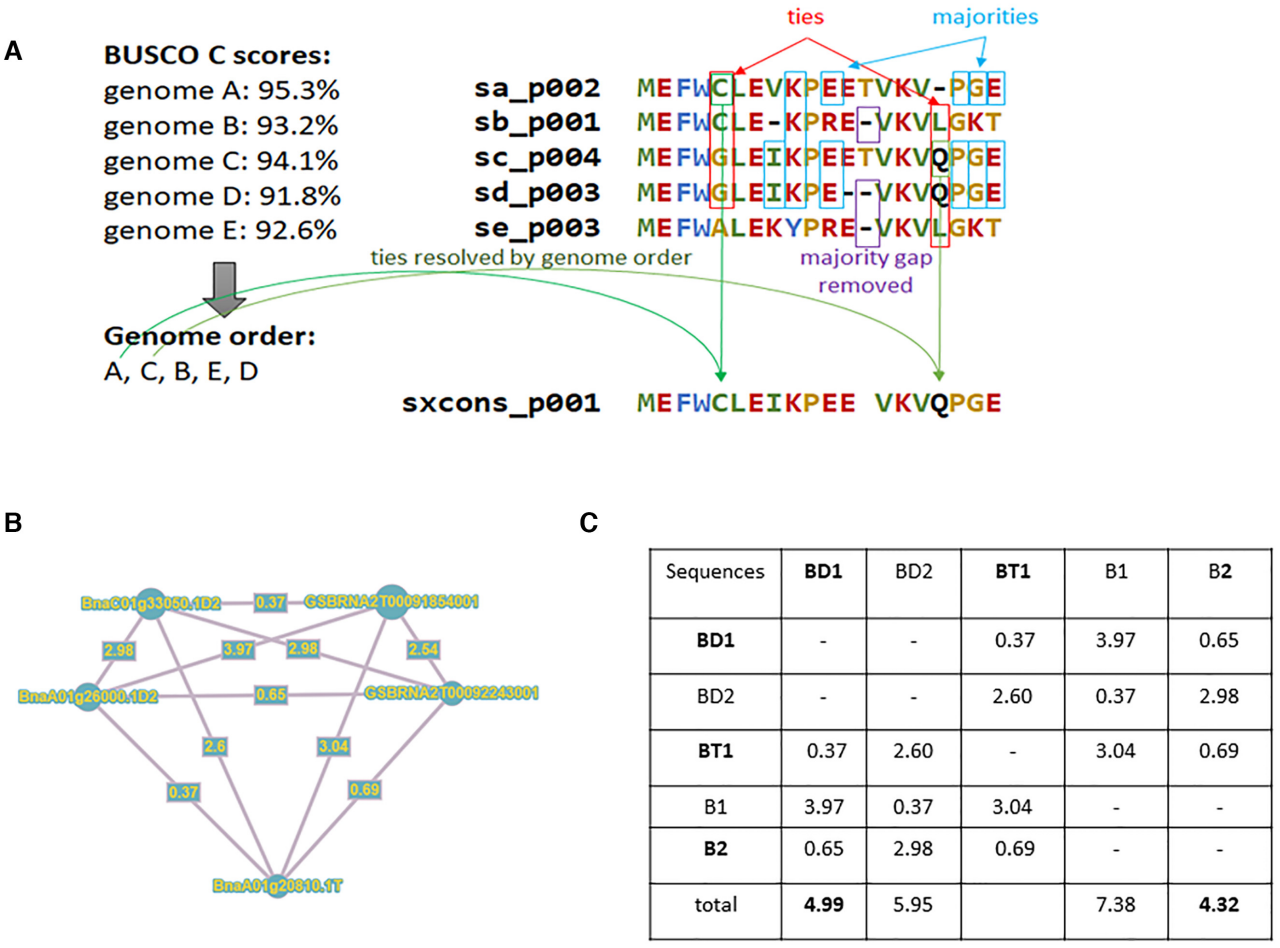
Pangene construction. (**A**) Schema illustrating the creation of consensus sequences. (**B**) Example of distance matrix network with five sequences from three genomes of *Brassica napus* (brana_pan_p029014). (**C**) Selection of representative sequences based on the minimum value of summed genetic distances. Sequences conserved are in bold.

Out of the full dataset, 105 genomes were considered to produce 19 pangenes computed with the get_homologues-est software v20092018 (21) (Table 1), based on NCBI Blast-v2.2 and using the following program options (-M -F -t 0 -m cluster) as previously applied to the *Brachypodium distachyon* dataset (13). We processed each cluster the following way:

- For single-gene copy clusters (a single sequence per genome), protein sequences were aligned using MAFFT v7.313 (22) (parameters adjusted according to the number of sequences) and an automatic procedure to generate a consensus sequence was applied (Figure 1A). For each position of the alignment, we kept the most frequent amino acid. In case of a tie, the amino acid of the genome with the highest BUSCO scores (‘complete’ then ‘fragmented’ and finally lowest ‘missing’ scores) was selected. Finally, if more gaps than amino acids were present, this position was removed from the sequence.
- For multi-copy clusters (multiple sequences per genome), we applied the same procedure as for single-gene copy clusters but added a preliminary step to select a representative sequence by cluster. Multiple sequence alignments were used to generate a distance matrix using distmat (Jukes-Cantor correction method) from EMBOSS v6.6 (Figure 1B). The matrix was required to define the distance for each sequence of all other genomes and the sequences with the smallest sum of distance was selected as representative of the considered genome (Figure 1C). Then, the consensus step was applied. It is worth mentioning that get_homologs-est generated sequence clusters of not too distantly related sequences. Large gene families can include several multi-copy clusters being grouped together at the sequence clustering step.
- For genotype-specific clusters (paralogs in a single genome), we generated a distance matrix between all sequences and the sequence with the lowest average distance (min(d/sum(d))) of all sequences was putatively considered as the most representative sequence. Those sequences were added to the pangene.
- Finally, singletons (cluster of one sequence) were searched for similarity using DIAMOND (23) with a default *e*-value on the protein-coding genes of all other species genomes to predict their putative prediction accuracy. Sequences with a minimum of one hit in at least two species were added to the pangene; otherwise sequences were excluded.

**Table 1.** List of GreenPhylDB pangenes with associated statistics

Species Code	Genomes available by species name	# Genes	Busco complete (%)	# Pangenes	% singletons (not in pangene)
**BRADI**	*Brachypodium distachyon (54 genomes)*	44 858 (average)	95.5 (average)	61 622	0.8
**BRANA**	*Brassica napus*	101 040	98.3	77 456	5.0
		80 382	96.2		0.5
		70 162	91.2		0.6
**BRAOL**	*Brassica* *oleracea*	35 400	80.7	60 869	2.4
		61 279	96.9		7.7
		56 687	99.5		3.3
**BRARR**	*Brassica rapa*	46 250	98.1	49 916	4.4
		46 721	97		4.1
**CAPAN**	*Capsicum annuum*	35 336	90.6	41 828	7.4
		34 476	84.6		6.0
		35 884	88.9		8.2
**CICAR**	*Cicer arietinum*	28 269	94.8	25 013	7.5
		30 257	93		11.7
**COCNU**	*Cocos nucifiera*	52 931	87.8	38 584	1.4
		34 953	86.3		7.0
**CUCSA**	*Cucumis sativus*	22 324	89.5	23 446	11.2
		23 780	94.8		7.5
		22 935	94.8		5.7
**IPOTF**	*Ipomoea trifida*	32 301	96.6	21 417	8.0
		30 227	94.7		5.3
**MAIZE**	*Zea mays*	39 591	94.6	45 301	4.6
		40 003	92.6		6.7
		40 557	87.3		9.4
		36 509	87.8		4.9
**MALDO**	*Malus domestica*	45 116	98.3	54 987	7.5
		95 232	91		24.5
		44 677	95.2		15.8
**MEDTR**	*Medicago truncatula*	50 444	96.6	43 859	19.2
		44 623	98.8		4.7
**MUSAC**	*Musa acuminata*	35 276	98.5	45905	5.5
		44 702	60.3		17.5
		32 692	71.2		18.2
		45 069	71.9		21.0
**ORYSA**	*Oryza sativa*	55 986	95.1	56785	11.5
		36 140	87.8		11.3
		37 549	96.1		11.1
		60 897	93.7		9.9
		60 123	89.4		10.7
		35 495	89.9		5.1
		35 594	99.3		2.3
**SORBI**	*Sorghum bicolor*	34 129	99.2	45054	11.8
		36 110	97.4		15.4
**SOYBN**	*Glycine max*	54 175	99.5	34512	5.0
		52 130	99.3		4.1
**THECC**	*Theobroma cacao*	21 330	99.2	32917	1.9
		44 607	99.6		23.1
**TRITU**	*Triticum turgidum*	107 891	99.6	54687	8.6
		67 182	98.9		5.3
**VITVI**	*Vitis vinifera*	41 733	98.4	43766	22.8
		96 331	92.4		5.1
		73 109	95.9		5.6

As a unique identifier was required for each pangene, we defined a nomenclature with a prefix composed of the [5-letter UniProt taxonomy database code]_pan, followed by p (for protein) and an auto-increment of 6-digits (e.g. musac_pan_p029014 for *Musa acuminata*).

### Sequence clustering and functional annotation

Pangenes and protein-coding genes of reference genomes (without pangenes) were searched all against all using DIAMOND. We then performed a clustering using TribeMCL (24) (*M* = 1.2, 2, 3 and 5), defining 4 levels of stringency (from 1 to 4) to take into account potential sub-classification and we obtained 9419, 18 805, 23 409 and 29 345 clusters, respectively.

We then scanned all sequences for protein domain signatures using InterProscan (25,26) and also crossed linked matches with UniProtKB-SwissProt entries (27). Cluster names resulting from curation from previous GreenPhylDB versions (2,28) were transferred when at least 51% of sequences were found clustered together as before (based on species in common between releases). In addition, for this release, we implemented an automatic method to name clusters based on the name of InterPro domains (family type only) that were found specific to clusters. In other words, when detected in at least 51% of the sequences composing an unannotated cluster, the name of the InterPro signature was assigned to it. In total, GreenPhylDB comprises 3538 clusters functionally characterized across the four levels.

### Homology inference

The previous phylogenetic-based methodology that we applied in the previous version has been conserved but uses a larger set of genomes to update our automated pipeline. The pipeline uses MAFFT for the multiple alignment step. FastTree 2 (v2.1.11) (29) was preferred over PhyML (30) due to the size of the clusters. Gene rooting and orthologous scoring was computed with Rap-Green (31) using the *viridiplantae* species tree extracted from NCBI taxonomy and converted into PhyloXML (2). We successfully produced gene trees at level 1 for more than 99.8% of the clusters (*n* = 9413) which enabled us to predict ∼17.8 million of orthologs and ∼1.8 million of in-paralogs (or ultra-paralogs) relationships. The pipeline was complemented by a Reciprocal Best Hits (RBH) method—computed between all pairs of genomes—that resulted in more than ∼12.1 million orthologous relationships.

## USING GREENPHYL

With this updated version, the website has received a face-lift. It now takes advantage of the bootstrap and D3.js frameworks to improve the user experience and to be more responsive. Alternatively, it can also be accessed programmatically using Resource Description Framework (RDF) as implemented in AgroLD, a knowledge-based system relying on semantic web technologies (32).

### Gene family pages

**Figure 2.**
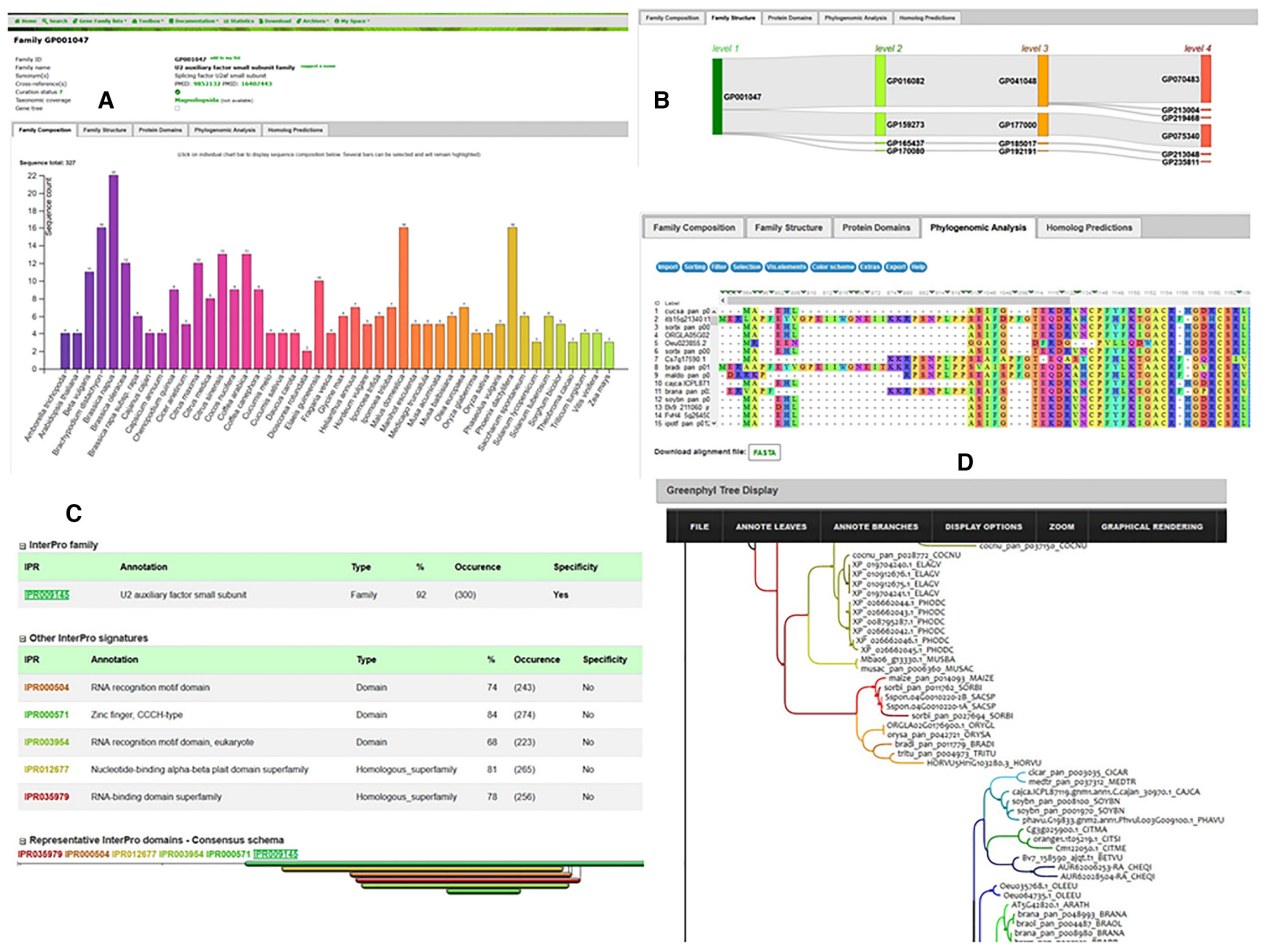
Overview of gene family interfaces for GP001047: U2 auxiliary factor small subunit family. (**A**) diagram of the sequence count (327) by species (46) (**B**) Sequence flow in cluster structure (from level 1 to 4). (**C**) InterPro domain specificity statistics. About 92% of sequences have the U2 auxiliary factor small subunit which is uniquely found in this cluster. (**D**) Viewers for multiple sequence alignment (MSAViewer) and phylogenetic tree (InTreeGreat).

All cluster (or gene family) pages present the same type of information divided into several tabs :

- *Gene family composition: a* bar chart allows users to visualize at a glance the composition of the gene family by species (Figure 2A). Species are ordered taxonomically to easily detect possible variations between phyla. Each bar is clickable and produces a table with the list of sequences and associated cross-references (i.e. InterPro, UniProt). Sequences can be exported in multiple formats and/or stored in a user list.
- *Gene family structure:* sequences are clustered at four levels of clustering, from less stringent to more stringent, in most cases narrowing the number of sequences (Figure 2B).
- *Protein domains:* here, InterProscan was used to assess the domain conservation consistency and the specificity of the sequence clusters (Figure 2C). For each cluster, we performed statistical analyses to determine whether InterPro signatures were specific and therefore not found in any other sequences of clusters of the same level.
- *Phylogenomic analyses:* this section includes multiple sequence alignments that can be downloaded and visualised using MSAviewer (33) and gene trees can be explored with InTreeGreat (https://www.southgreen.fr/content/intreegreat-tool) and PhyD3 (34) (Figure 2D). Some gene trees can be very large, and the interface proposes an option to prune automatically the tree based on user choice for a range of species.
- *Homologous predictions:* the interface enables users to display and refine all the homologies detected by the phylogenetic-based approach and Reciprocal Best Hits (RBH). It is possible to filter and select only a subset of species of interest.

### Pangene pages

**Figure 3.**
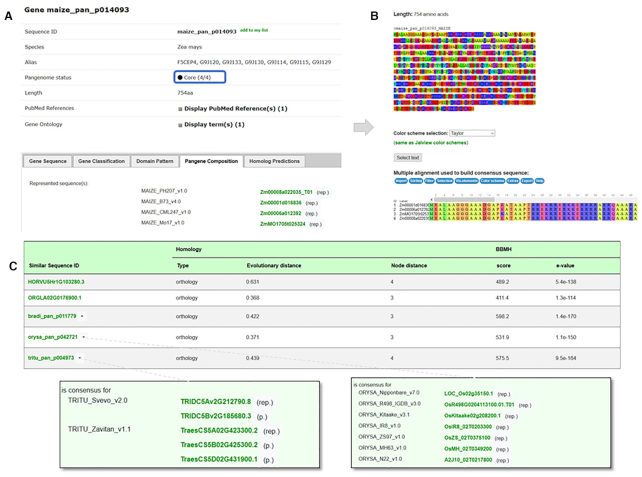
Example of a pangene page (i.e. maize_pan_p014093) member of the U2 auxiliary factor small subunit gene family (GP001047). (**A**) Gene composition tab: all genes are part of core compartment for those four genomes since a representative sequence exists for each of the reference genomes. (**B**) Consensus sequences and associated multiple alignment. (**C**) List of homologs: the *Zea mays* pangene is predicted orthologous to pangenes in *Triticum turgidum* and in *Oryza sativa*. The Popups (green rectangles) display the sequence compositions of the respective pangenes. (.rep) refers to the representative sequence kept to create the consensus and (.p) to paralog sequences not used in the consensus.

The new page type for pangene sequences is a central and unique concept in this version as they were used for the clustering and homology predictions instead of all individual sequences that compose it. When browsing these pages, users can quickly see which genes are present or missing by looking at the status: core or dispensable compartments. Then, information related to the sequence composition is reported. Users can access information about pangene classification, the consensus sequence (except for singletons) with the multiple sequence alignments used to create it as well as related homology predictions (Figure 3). In the case of multi-copy clusters, it is possible to see which sequence was selected as representative (.rep) or participant (.p) and also why they were selected by browsing the distance matrix.

### New tools

The database can still be searched via keyword searches or by entering a query sequence for similarity search using DIAMOND (which replaces BLAST for faster processing), enhanced by new tools to further explore those datasets.

#### Quick search

A new interface has been designed to retrieve in a comprehensive and concise way all the information associated with gene family names, sequence annotation and annotations from InterPro and UniProt mappings.

#### IPR2genomes—InterPro domain search

It is now possible to search sequences and associated clusters based on a combination of InterPro domain signatures. This can be particularly helpful when searching for transcription factors for which sequences must contain some domains but not others (35) as the Markov Cluster Algorithm (MCL) may fail grouping them accurately. The interface allows the use of various operators (e.g. AND, OR, NOT, ONLY) to filter a set of sequences for all genomes. Results can be compared with the MCL automatic clustering to check consistencies or differences.

#### TreePattern —Tree topology search

A tree search can be done by filtering on gene tree topologies (31). Users can draw the topology with species or taxonomic groups as leaves or nodes of the tree and apply constraints such presence or absence of duplications. Resulting trees can be accessed individually (or exported in bulk results as CSV file) and defined patterns are highlighted. This feature is useful for identifying gene families with an expected evolutionary scenario due to gene duplications.

### Manual curation and sharing of gene families

While automatic clustering is a relevant and efficient starting point, sometimes limitations (e.g. missing sequences, errors in gene annotations) are present and prevent access to ready-to-use datasets, justifying a deeper characterization that will eventually lead to a refinement of the automatic clustering. As a result, knowledge generated on individual gene families is often available only in publications and their supplementary information as PDFs. To encourage knowledge capture, we developed a section for advanced users to create and share their own gene families. Two methods are possible: users can either start from scratch and upload their data or use existing clusters and take advantage of multiples operations implemented in the ‘MyList’ features: such feature was indeed developed to facilitate intersecting, combining clusters. This new tool can be valuable during the review process by providing a unique identifier to referees—and eventually to users—to explore the structure and composition of the submitted gene family.

## USE CASES

In this section, we describe three possible uses that are enabled by this new GreenPhylDB version. Concrete examples related to each of them are further documented in Supplementary Data.

- You want to analyse a gene family with focus on a specific species sampling.
  - search the gene family by keyword(s), locus gene ids or sequences using dedicated search families (toolbox menu).
  - browse the family structure and explore sub clusters at level 2, 3 or 4.
  - browse the family composition of the cluster (or a specific sub cluster) to list all the sequences and pangenes selecting one or several bars in the diagram. Export the selected sequences of interest using proposed file formats.
  - (optional) if the gene family sequences are characterized by several protein domains, check possible additional sequences in the database using IPR2genomes (toolbox menu). For individual protein domains, its specificity is indicated in each gene family page (if specific, no need to search in other gene families).
  - (optional) In case of additional analyses (e.g. addition of sequences from a new sequences genomes), you can create a ‘custom family’ by uploading the sequences on the website and share the link in a manuscript, with collaborators or reviewers during the review process for a user-friendly exploration of the dataset.
- You are interested in finding genes that are linked to a specific evolutionary scenario (e.g. duplicated genes in one species but not in another)
  - retrieve full list of gene trees and related gene families using TreePattern (toolbox menu).
  - browse examples gene families to see patterns (highlighted with dashed lines in the tree).
  - click on the sequence name to access the family.
  - browse the family composition of the cluster (or a specific sub cluster) to list all the sequences and pangenes selecting one or several bars in the diagram. Export the selected sequences of interest using proposed file formats
- You have a candidate gene in rice, maize or banana (or any of the 19 pangenes) and want to retrieve the related sequences in other genomes of the same species and then find orthologs in other species (e.g. *Arabidopsis*).
  - search by sequence or by locus ID to identify the pangene ID.
  - retrieve the pangene composition to get all members and check the status (core or dispensable).
  - (optional) check the multiple gene alignment to see level of divergence.
  - go to the gene family and explore (or download) the gene tree.
  - retrieve predicted orthologs (by phylogeny and/or Reciprocal Best Hits). Alternatively, use the homologous sequence search directly (toolbox menu).

## CONCLUSION

This new version of GreenPhylDB provides a unique way to scale up plant comparative genomics studies across multiple plants species by leveraging pangenomic datasets. This release paves the way to the transition from reference-based genomics to pangenome-based systems and tools. In this context, the website includes new powerful search interfaces to explore the content of the gene family collection. Advanced users can also deposit the results of their expert gene family curation for further use and reference. GreenPhylDB is an important resource to understand the genetic basis of genome diversity among plant species and has the potential to accelerate gene discovery to support crop improvement.

## DATA AVAILABILITY

All datasets produced by our automatic analyses are accessible via GreenPhylDB user interfaces or can be downloaded at https://www.greenphyl.org/cgi-bin/downloads.cgi.

## Supplementary Material

gkaa1068_Supplemental_File

## References

[1] Van Bel M., Diels T., Vancaester E., Kreft L., Botzki A., Van de Peer Y., Coppens F., Vandepoele K. PLAZA 4.0: an integrative resource for functional, evolutionary and comparative plant genomics. Nucleic Acids Res. 2018; 46:D1190–D1196. 29069403 10.1093/nar/gkx1002 PMC5753339

[2] Rouard M., Guignon V., Aluome C., Laporte M.-A., Droc G., Walde C., Zmasek C.M., Périn C., Conte M.G. GreenPhylDB v2.0: comparative and functional genomics in plants. Nucleic Acids Res. 2011; 39:D1095–D1102. 20864446 10.1093/nar/gkq811 PMC3013755

[3] Goodstein D.M., Shu S., Howson R., Neupane R., Hayes R.D., Fazo J., Mitros T., Dirks W., Hellsten U., Putnam N. et al. Phytozome: a comparative platform for green plant genomics. Nucleic Acids Res. 2012; 40:D1178–D1186. 22110026 10.1093/nar/gkr944 PMC3245001

[4] Gupta P., Naithani S., Tello-Ruiz M.K., Chougule K., D’Eustachio P., Fabregat A., Jiao Y., Keays M., Lee Y.K., Kumari S. et al. Gramene database: navigating plant comparative genomics resources. Curr. Plant Biol. 2016; 7–8:10–15. 10.1016/j.cpb.2016.12.005 PMC5509230 28713666

[5] Bolser D., Staines D.M., Pritchard E., Kersey P. Edwards D. Ensembl plants: integrating tools for visualizing, mining, and analyzing plant genomics data. Plant Bioinformatics: Methods and Protocols, Methods in Molecular Biology. 2016; NY Springer 115–140. 10.1007/978-1-4939-3167-5_6 26519403

[6] Golicz A.A., Batley J., Edwards D. Towards plant pangenomics. Plant Biotechnol. J. 2016; 14:1099–1105. 26593040 10.1111/pbi.12499 PMC11388911

[7] Tranchant‐Dubreuil C., Rouard M., Sabot F. Roberts J.A. Plant pangenome: impacts on phenotypes and evolution. Annual Plant Reviews Online. 2019; 453–478.

[8] Marschall T., Marz M., Abeel T., Dijkstra L., Dutilh B.E., Ghaffaari A., Kersey P., Kloosterman W.P., Mäkinen V., Novak A.M. et al. Computational pan-genomics: status, promises and challenges. Brief. Bioinform. 2018; 19:118–135. 27769991 10.1093/bib/bbw089 PMC5862344

[9] Tao Y., Zhao X., Mace E., Henry R., Jordan D. Exploring and exploiting pan-genomics for crop improvement. Mol. Plant. 2019; 12:156–169. 30594655 10.1016/j.molp.2018.12.016

[10] Gabur I., Chawla H.S., Snowdon R.J., Parkin I.A.P. Connecting genome structural variation with complex traits in crop plants. Theor. Appl. Genet. 2019; 132:733–750. 30448864 10.1007/s00122-018-3233-0

[11] Golicz A.A., Bayer P.E., Barker G.C., Edger P.P., Kim H., Martinez P.A., Chan C.K.K., Severn-Ellis A., McCombie W.R., Parkin I.A.P. et al. The pangenome of an agronomically important crop plant Brassica oleracea. Nat. Commun. 2016; 7:13390. 27834372 10.1038/ncomms13390 PMC5114598

[12] Montenegro J.D., Golicz A.A., Bayer P.E., Hurgobin B., Lee H., Chan C.-K.K., Visendi P., Lai K., Doležel J., Batley J. et al. The pangenome of hexaploid bread wheat. Plant J. 2017; 90:1007–1013. 28231383 10.1111/tpj.13515

[13] Gordon S.P., Contreras-Moreira B., Woods D.P., Marais D.L.D., Burgess D., Shu S., Stritt C., Roulin A.C., Schackwitz W., Tyler L. et al. Extensive gene content variation in the Brachypodium distachyon pan-genome correlates with population structure. Nat. Commun. 2017; 8:2184. 29259172 10.1038/s41467-017-02292-8 PMC5736591

[14] Wang W., Mauleon R., Hu Z., Chebotarov D., Tai S., Wu Z., Li M., Zheng T., Fuentes R.R., Zhang F. et al. Genomic variation in 3,010 diverse accessions of Asian cultivated rice. Nature. 2018; 557:43–49. 29695866 10.1038/s41586-018-0063-9 PMC6784863

[15] Zhao Q., Feng Q., Lu H., Li Y., Wang A., Tian Q., Zhan Q., Lu Y., Zhang L., Huang T. et al. Pan-genome analysis highlights the extent of genomic variation in cultivated and wild rice. Nat. Genet. 2018; 50:278–284. 29335547 10.1038/s41588-018-0041-z

[16] Hirsch C.N., Foerster J.M., Johnson J.M., Sekhon R.S., Muttoni G., Vaillancourt B., Peñagaricano F., Lindquist E., Pedraza M.A., Barry K. et al. Insights into the maize pan-genome and pan-transcriptome. Plant Cell. 2014; 26:121–135. 24488960 10.1105/tpc.113.119982 PMC3963563

[17] Gao L., Gonda I., Sun H., Ma Q., Bao K., Tieman D.M., Burzynski-Chang E.A., Fish T.L., Stromberg K.A., Sacks G.L. et al. The tomato pan-genome uncovers new genes and a rare allele regulating fruit flavor. Nat. Genet. 2019; 51:1044–1051. 31086351 10.1038/s41588-019-0410-2

[18] Hübner S., Bercovich N., Todesco M., Mandel J.R., Odenheimer J., Ziegler E., Lee J.S., Baute G.J., Owens G.L., Grassa C.J. et al. Sunflower pan-genome analysis shows that hybridization altered gene content and disease resistance. Nat. Plants. 2019; 5:54–62. 30598532 10.1038/s41477-018-0329-0

[19] Koonin E.V. Orthologs, paralogs and evolutionary genomics. Annu. Rev. Genet. 2005; 39:309–338. 16285863 10.1146/annurev.genet.39.073003.114725

[20] Simão F.A., Waterhouse R.M., Ioannidis P., Kriventseva E.V., Zdobnov E.M. BUSCO: assessing genome assembly and annotation completeness with single-copy orthologs. Bioinformatics. 2015; 31:3210–3212. 26059717 10.1093/bioinformatics/btv351

[21] Contreras-Moreira B., Cantalapiedra C.P., García-Pereira M.J., Gordon S.P., Vogel J.P., Igartua E., Casas A.M., Vinuesa P. Analysis of plant pan-genomes and transcriptomes with GET_HOMOLOGUES-EST, a clustering solution for sequences of the same species. Front. Plant Sci. 2017; 8:184. 28261241 10.3389/fpls.2017.00184 PMC5306281

[22] Katoh K., Standley D.M. MAFFT multiple sequence alignment software version 7: improvements in performance and usability. Mol. Biol. Evol. 2013; 30:772–780. 23329690 10.1093/molbev/mst010 PMC3603318

[23] Buchfink B., Xie C., Huson D.H. Fast and sensitive protein alignment using DIAMOND. Nat. Methods. 2015; 12:59–60. 25402007 10.1038/nmeth.3176

[24] Enright A.J., Van Dongen S., Ouzounis C.A. An efficient algorithm for large-scale detection of protein families. Nucleic Acids Res. 2002; 30:1575–1584. 11917018 10.1093/nar/30.7.1575 PMC101833

[25] Zdobnov E.M., Apweiler R. InterProScan—an integration platform for the signature-recognition methods in InterPro. Bioinformatics. 2001; 17:847–848. 11590104 10.1093/bioinformatics/17.9.847

[26] Mitchell A.L., Attwood T.K., Babbitt P.C., Blum M., Bork P., Bridge A., Brown S.D., Chang H.-Y., El-Gebali S., Fraser M.I. et al. InterPro in 2019: improving coverage, classification and access to protein sequence annotations. Nucleic Acids Res. 2019; 47:D351–D360. 30398656 10.1093/nar/gky1100 PMC6323941

[27] Magrane M., Consortium U. UniProt Knowledgebase: a hub of integrated protein data. Database (Oxford). 2011; 2011:bar009. 21447597 10.1093/database/bar009 PMC3070428

[28] Conte M.G., Gaillard S., Lanau N., Rouard M., Périn C. GreenPhylDB: a database for plant comparative genomics. Nucleic Acids Res. 2008; 36:D991–D998. 17986457 10.1093/nar/gkm934 PMC2238940

[29] Price M.N., Dehal P.S., Arkin A.P. FastTree 2—approximately maximum-likelihood trees for large alignments. PLoS One. 2010; 5:e9490. 20224823 10.1371/journal.pone.0009490 PMC2835736

[30] Guindon S., Delsuc F., Dufayard J.-F., Gascuel O. Estimating maximum likelihood phylogenies with PhyML. Methods Mol. Biol. 2009; 537:113–137. 19378142 10.1007/978-1-59745-251-9_6

[31] Dufayard J.-F., Duret L., Penel S., Gouy M., Rechenmann F., Perrière G. Tree pattern matching in phylogenetic trees: automatic search for orthologs or paralogs in homologous gene sequence databases. Bioinformatics. 2005; 21:2596–2603. 15713731 10.1093/bioinformatics/bti325

[32] Venkatesan A., Ngompe G.T., Hassouni N.E., Chentli I., Guignon V., Jonquet C., Ruiz M., Larmande P. Agronomic Linked Data (AgroLD): a knowledge-based system to enable integrative biology in agronomy. PLoS One. 2018; 13:e0198270. 30500839 10.1371/journal.pone.0198270 PMC6269127

[33] Yachdav G., Wilzbach S., Rauscher B., Sheridan R., Sillitoe I., Procter J., Lewis S.E., Rost B., Goldberg T. MSAViewer: interactive JavaScript visualization of multiple sequence alignments. Bioinformatics. 2016; 32:3501–3503. 27412096 10.1093/bioinformatics/btw474 PMC5181560

[34] Kreft L., Botzki A., Coppens F., Vandepoele K., Van Bel M. PhyD3: a phylogenetic tree viewer with extended phyloXML support for functional genomics data visualization. Bioinformatics. 2017; 33:2946–2947. 28525531 10.1093/bioinformatics/btx324

[35] Lang D., Weiche B., Timmerhaus G., Richardt S., Riano-Pachon D.M., Correa L.G.G., Reski R., Mueller-Roeber B., Rensing S.A. Genome-wide phylogenetic comparative analysis of plant transcriptional regulation: a timeline of loss, gain, expansion, and correlation with complexity. Genome. Biol. Evol. 2010; 2:488–503. 20644220 10.1093/gbe/evq032 PMC2997552

