# Correction to ‘GreenPhylDB v5: a comparative pangenomic database for plant genomes’

**DOI:** 10.1093/nar/gkab564

**Published:** 2021-06-23

**Authors:** Valentin Guignon, Abdel Toure, Gaëtan Droc, Jean-François Dufayard, Matthieu Conte, Mathieu Rouard

**Affiliations:** Bioversity International, Parc Scientifique Agropolis II, 34397 Montpellier, France; French Institute of Bioinformatics (IFB)––South Green Bioinformatics Platform, Bioversity, CIRAD, INRAE, IRD, F-34398 Montpellier, France; Syngenta Seeds SAS, 31790 Saint-Sauveur, France; French Institute of Bioinformatics (IFB)––South Green Bioinformatics Platform, Bioversity, CIRAD, INRAE, IRD, F-34398 Montpellier, France; AGAP, Univ de Montpellier, CIRAD, INRAE, Montpellier SupAgro, F-34398 Montpellier, France; CIRAD, UMR AGAP, F-34398 Montpellier, France; French Institute of Bioinformatics (IFB)––South Green Bioinformatics Platform, Bioversity, CIRAD, INRAE, IRD, F-34398 Montpellier, France; AGAP, Univ de Montpellier, CIRAD, INRAE, Montpellier SupAgro, F-34398 Montpellier, France; CIRAD, UMR AGAP, F-34398 Montpellier, France; Syngenta Seeds SAS, 31790 Saint-Sauveur, France; Bioversity International, Parc Scientifique Agropolis II, 34397 Montpellier, France; French Institute of Bioinformatics (IFB)––South Green Bioinformatics Platform, Bioversity, CIRAD, INRAE, IRD, F-34398 Montpellier, France

The author names were typeset as supplied by the authors in their manuscript and were approved in proofs. However, the authors wish to correct the author list in their article (1) because the first and last names were inverted.

This change does not affect the results, discussion and conclusions presented in the article. The published article has been updated.

## References

[1] Guignon V., Toure A., Droc G., Dufayard J-.F., Conte M., Rouard M. GreenPhylDB v5: a comparative pangenomic database for plant genomes. Nucleic Acids Res. 2021; 49:D1464–D1471. 33237299 10.1093/nar/gkaa1068 PMC7779052

