# Supplementary material for: GreenPhylDB v5: a comparative pangenomic database for plant genomes
Source: Nucleic Acids Res. 2020 Nov 25;49(D1):D1464–71. doi: 10.1093/nar/gkaa1068 (PMC7779052; doi:10.1093/nar/gkaa1068)

# SUPPLEMENTARY MATERIAL

## Supplementary Table S1. List and sources of species and public datasets used in GreenPhyl v5

| **Species** | **Reference** | **Data Source** | **# genes** |
| --- | --- | --- | --- |
| *Amborella trichopoda* | Albert et al, 2013 | amborella.org | 26846 |
| *Arabidopsis thaliana* | AGI, 2000 | ARAPORT | 27655 |
| *Beta vulgaris* | Minoche et al. 2015 | The Beta vulgaris Resource | 29088 |
| *Brachypodium distachyon* | Gordon et al, 2017 | Brachypan | 44858 |
| *Brassica napus* | Chalhoub et al, 2014. Science | Brassicadb | 101040 |
| *Brassica napus* | Bayer et al, 2017 | appliedbioinformatics.com.au | 80382 |
| *Brassica napus* | Bayer et al, 2017 | appliedbioinformatics.com.au | 70162 |
| *Brassica oleracea* | Liu et al, 2014 | BolBase | 35400 |
| *Brassica oleracea* | Belsner et al, 2018 | Genoscope | 61279 |
| *Brassica oleracea* | Parkin et al, 2014 | RefSeq | 56687 |
| *Brassica rapa* | Zhang et al, 2018 | brassicadb | 46250 |
| *Brassica rapa* | Belsner et al, 2018 | Genoscope | 46721 |
| *Cajanus cajan* | Varshney et al, 2012 | Legume Information System | 40071 |
| *Capsicum annuum* | Qin et al, 2014 | SOL Genomics | 35336 |
| *Capsicum annuum* | Qin et al, 2014 | SOL Genomics | 34476 |
| *Capsicum annuum* | Kim et al, 2014 | Pepper Genome v2.0 | 35884 |
| *Chenopodium quinoa* | Jarvis et al, 2017 | Phytozome | 44776 |
| *Cicer arietinum* | Varshney et al, 2013 | Legume Information System | 28269 |
| *Cicer arietinum* | Parween et al, 2015 | Legume Information System | 30257 |
| *Citrus maxima* | Wang et al, 2017 | Citrus sinensis Annotation Project | 30123 |
| *Citrus medica* | Wang et al, 2017 | Citrus sinensis Annotation Project | 46851 |
| *Citrus sinensis* | Xu et al, 2013 | Citrus sinensis Annotation Project | 29406 |
| *Cocos nucifera* | unpublished | Request to authors | 52931 |
| *Cocos nucifera* | Lantican et al, 2019 | Request to authors | 34953 |
| *Coffea arabica* | Scalabrin et al, 2020 | ILLYCAFFÈ AND LAVAZZA | 78311 |
| *Coffea canephora* | Denoeud et al, 2014 | Coffee Genome Hub | 25574 |
| *Cucumis melo* | Garcia-Mas et al. 2012 | CuGenDB | 29980 |
| *Cucumis sativus* | unpublished | CuGenDB | 22324 |
| *Cucumis sativus* | Huang et al, 2009 | EnsemBL | 23780 |
| *Cucumis sativus* | Qi et al, 2013 | CuGenDB | 22935 |
| *Daucus carota* | Iorizzo et al, 2016 | Phytozome | 32118 |
| *Dioscorea rotundata* | Tamiru et al, 2017 | IBRC website | 26198 |
| *Elaeis guineensis* | Singh et al, 2013 | NCBI RefSeq | 41887 |
| *Fragaria vesca* | ‎Shulaev et al, 2011 | GDR | 28588 |
| *Glycine max* | Schmutz et al, 2010 | Phytozome | 54175 |
| *Glycine max* | Shen et al, 2018 | Genome warehouse | 52130 |
| *Helianthus annuus* | Badouin et al, 2017 | Sunflower genome portal | 52243 |
| *Hordeum vulgare* | IBGC, 2012 | EnsemBL genome | 37705 |
| *Ipomoea trifida* | Wu et al, 2018 | Dryad | 32301 |
| *Ipomoea trifida* | Li et al, 2019 | Request to authors | 30227 |
| *Ipomoea triloba* | Wu et al, 2018 | Dryad | 31426 |
| *Malus domestica* | Daccord et al, 2017 | GDR | 45116 |
| *Malus domestica* | Li et al, 2016 | GDR | 95232 |
| *Malus domestica* | Zhang et al, 2019 | Github | 44677 |
| *Manihot esculenta* | Bredeson et al, 2016 | Phytozome | 33033 |
| *Medicago truncatula* | Tang et al, 2014 | Medicago Genome Database | 50444 |
| *Medicago truncatula* | Pecrix et al, 2018 | Medicago genome portal | 44623 |
| *Musa acuminata* | Martin et al, 2016 | Banana Genome Hub | 35276 |
| *Musa acuminata* | Rouard et al, 2018 | Banana Genome Hub | 44702 |
| *Musa acuminata* | Rouard et al, 2018 | Banana Genome Hub | 32692 |
| *Musa acuminata* | Rouard et al, 2018 | Banana Genome Hub | 45069 |
| *Musa balbisiana* | Wang et al, 2019 | Banana Genome Hub | 33021 |
| *Olea europaea* | Unver et al, 2017 | IOGC | 50684 |
| *Oryza glaberrima* | stein et al, 2018 | I-OMAP | 33164 |
| *Oryza sativa* | Goff et al, 2002 | MSU | 55986 |
| *Oryza sativa* | Stein et al, 2018 | IOMAP (Cyverse) | 36140 |
| *Oryza sativa* | Du et al, 2017 | MBKBase | 37549 |
| *Oryza sativa indica* | Zhang et al, 2017 | RIGW | 60897 |
| *Oryza sativa indica* | Zhang et al, 2017 | RIGW | 60123 |
| *Oryza sativa indica* | Stein et al, 2018 | IOMAP (Cyverse) | 35495 |
| *Oryza sativa japonica* | - | Phytozome | 35594 |
| *Phaseolus vulgaris* | Schmutz et al. 2014 | Legume Information System | 27433 |
| *Phoenix dactylifera* | Al-Mssallem et al, 2013 | NCBI refseq | 40634 |
| *Saccharum spontaneum* | Zhang et al, 2018 | The Ming Laboratory | 83826 |
| *Solanum lycopersicum* | ITAG | SOL Genomics | 34727 |
| *Solanum tuberosum* | Sharma, S. K. et al, 2013 | SOL Genomics | 39031 |
| *Sorghum bicolor* | McCormick RF, 2017 | JGI | 34129 |
| *Sorghum bicolor* | Deschamp et al, 2018 | Request to authors | 36110 |
| *Theobroma cacao* | Argout et al, 2018 | Cacao Genome Hub | 21330 |
| *Theobroma cacao* | Motamayor et al, 2013 | Cacao Genome Database | 44607 |
| *Triticum turgidum* | IWGSC | URGI | 107891 |
| *Triticum turgidum* | Maccaferri et al, 2019 | e!DAL | 67182 |
| *Vitis vinifera* | Canaguier et al, 2017 | URGI | 41733 |
| *Vitis vinifera* | Minio et al, 2018 | Cantu Lab | 96331 |
| *Vitis vinifera* | Minio et al, 2019 | Cantu Lab | 73109 |
| *Zea mays* | Schnable et al, 2009 | Gramene (release 60) | 39591 |
| *Zea mays* | Yang et al, 2017 | MMGDB | 40003 |
| *Zea mays* | Hirsch et al, 2016 | Dryad | 40557 |
| *Zea mays* | Lu et al, 2015 | MaizeGDB | 36509 |

## Use case 1: A user wants to study the NAC transcription factors, with emphases on Maize genomes.

- Enter “NAC” as keyword in the [Search](https://www.greenphyl.org/cgi-bin/quick_search.cgi).
- A list of 41 GreenPhyl (GP) families is returned ordered by number of hits in different categories (e.g. Gene family name, InterPro or GO description, Locus tags ids, Uniprot names and synonyms)
- The first result is the NAC transcription factor family. In this example, other clusters contain an InterPro domain with NAC in their description.


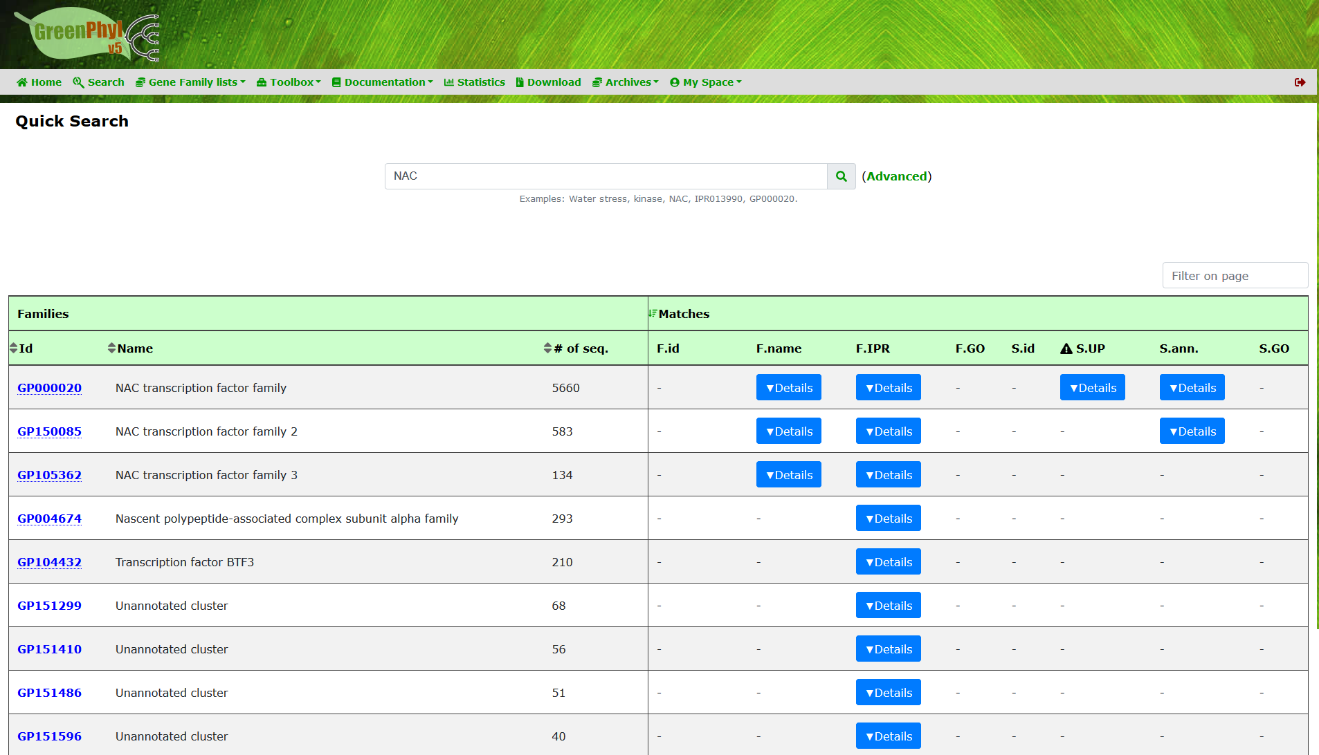


Some contains NAC domains that are irrelevant (e.g. [IPR002715](http://www.ebi.ac.uk/interpro/entry/IPR002715) or [IPR038187](http://www.ebi.ac.uk/interpro/entry/IPR038187)) to NAC transcription factors family but other have the relevant domains. The latter were not clustered in the main NAC family, likely due to higher sequence divergence that occurred in some clades. An example of this is [GP153044](https://www.greenphyl.org/cgi-bin/family.cgi?p=id&family_id=1102904#tab-famcomp) which includes only one or two sequences for Poaceae species or [GP153725](https://www.greenphyl.org/cgi-bin/family.cgi?p=id&family_id=1103745#tab-famcomp) for the Arecaceae.

- Alternatively, Look for Maize (*Zea mays*) pangenes with IPR corresponding to [IPR036093](http://www.ebi.ac.uk/interpro/entry/IPR036093): NAC domain superfamily and make sure they don’t have other irrelevant domains using this [IPR2Genome](https://www.greenphyl.org/cgi-bin/ipr2genomes.cgi).


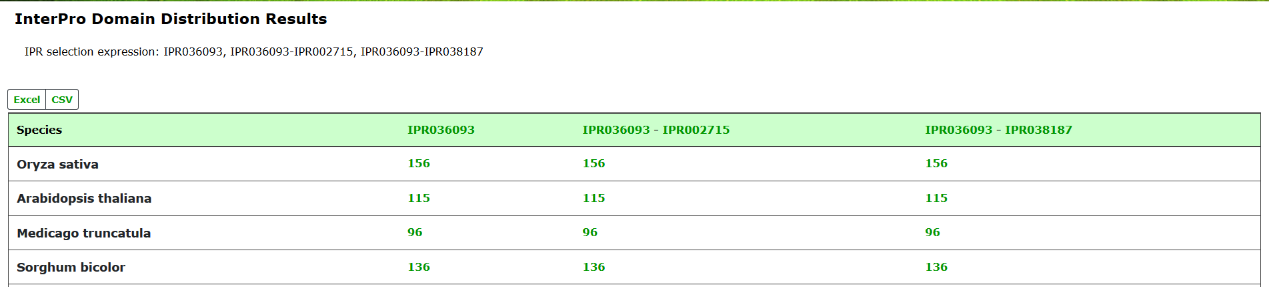


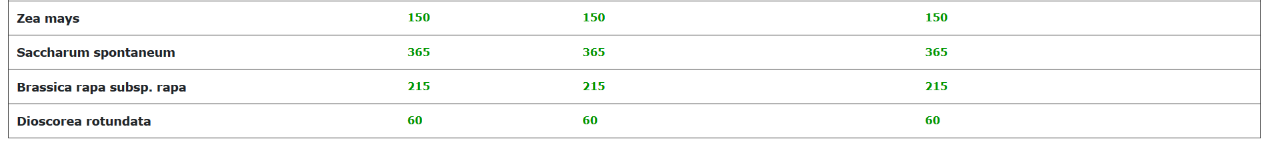


- The tool identified 150 pansequences that are distributed in 10 GP families listed hereunder. All sequences can be exported in FASTA format.


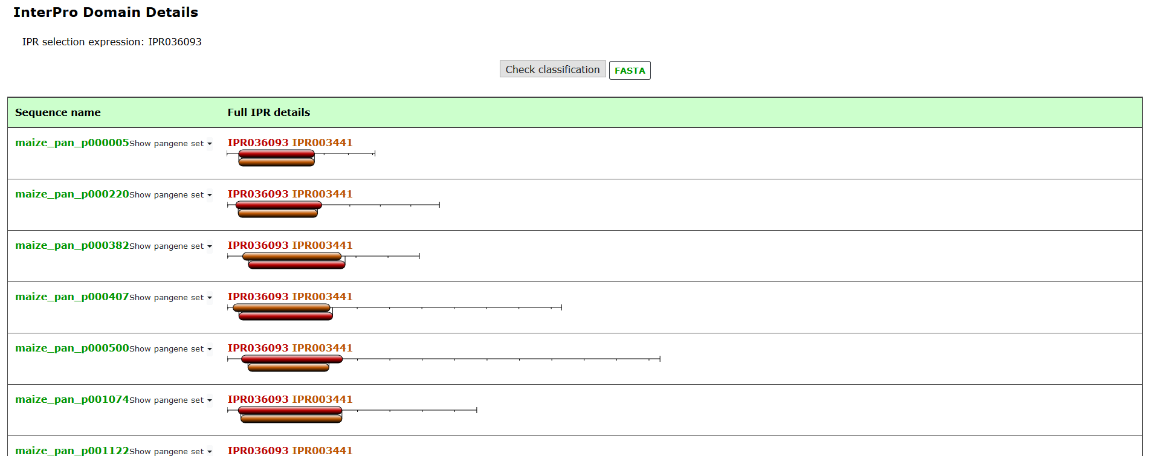


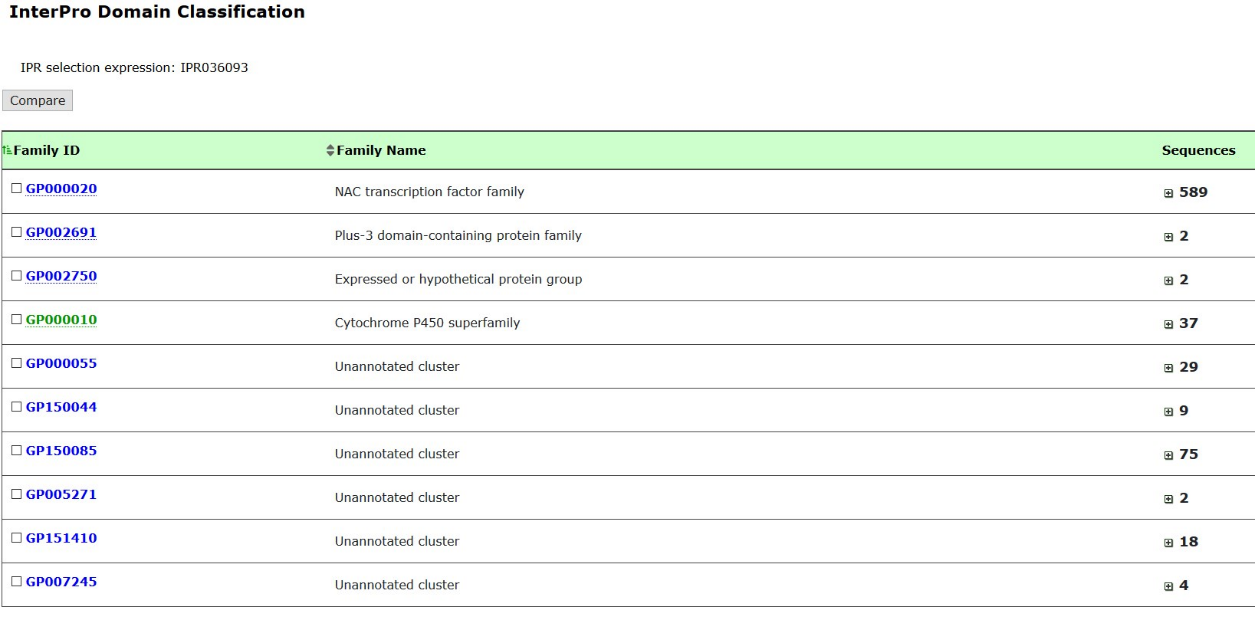


Now looking at the main NAC TF family [GP000020](https://www.greenphyl.org/cgi-bin/family.cgi?p=id&family_id=1095311), which contains 136 of the sequence (out of 150 pangenes previously identified).


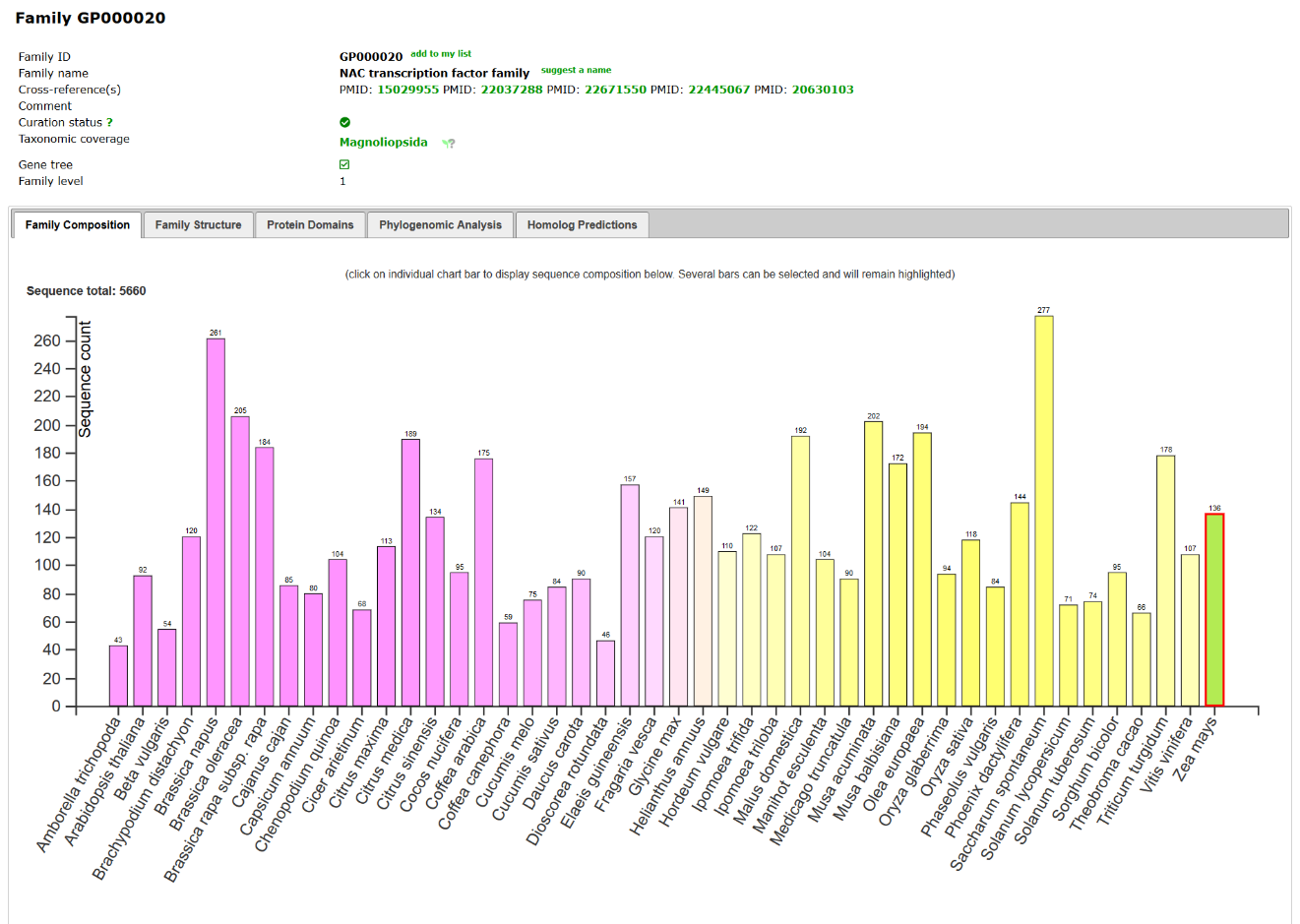


By clicking on the *Zea mays* bar, the list of pangenes are displayed under the graph. By clicking on other species, list of sequence is added to the table. Table information can be exported in Excel or CSV format while sequences can be exported in FASTA format.

Alternatively, the family or sequences can be stored in ‘My List’ which allows to perform a number of operations.


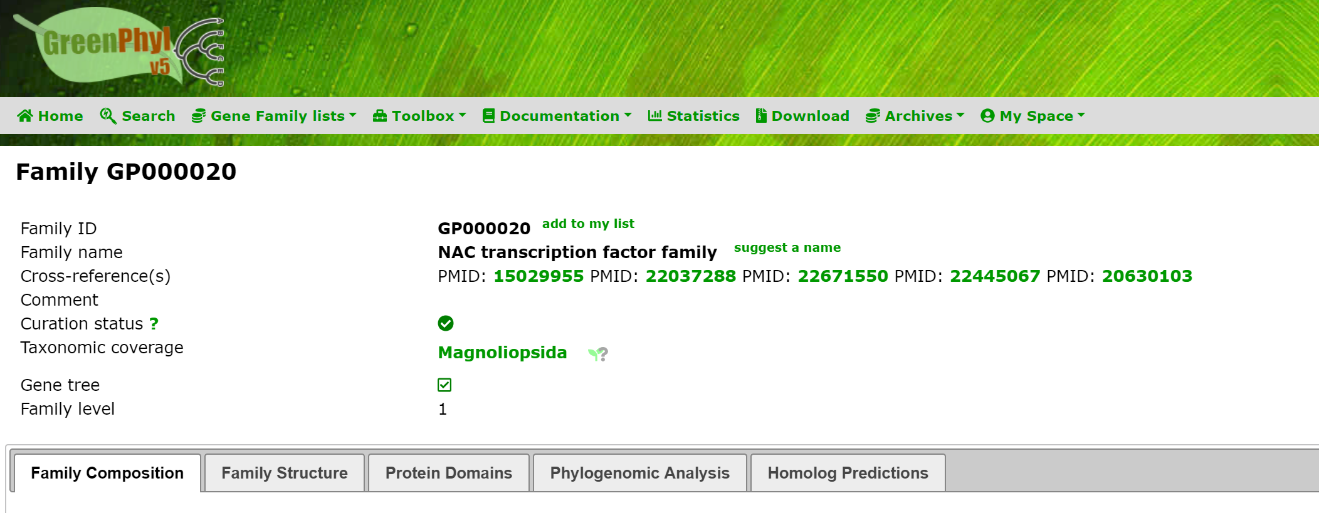


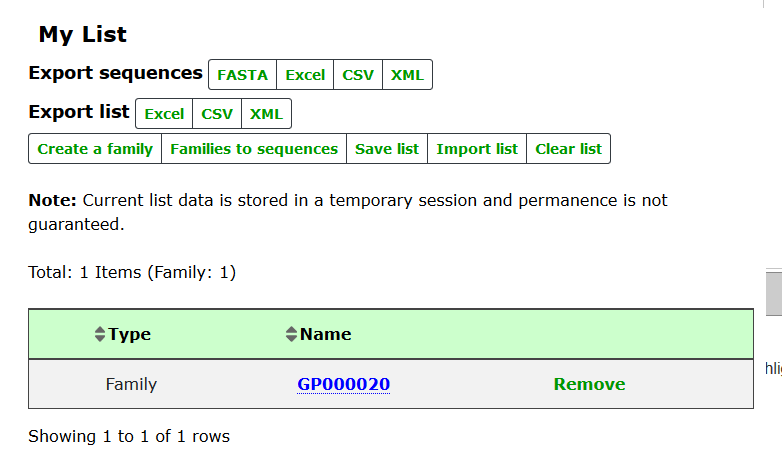


In the table below, the composition of a pangene can be unveiled at a glance by clicking on the
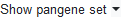


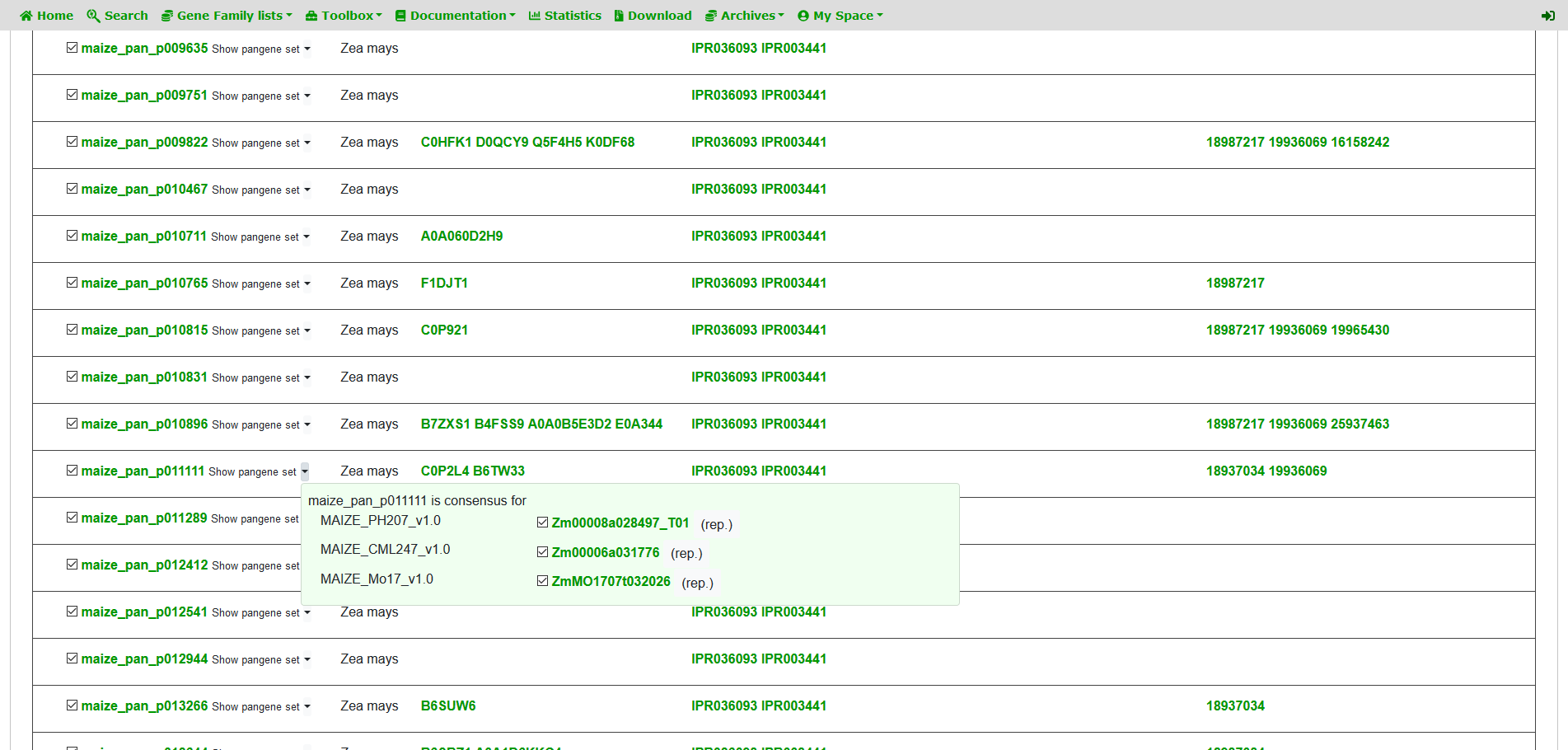


- The Maize pangenome is composed 4 genomes in GreenPhylDB (B73, Mo17, PH207 and CML247). Most of the pangenes are composed on 4 sequences, one for each genome (core), sometimes more with paralogs.
- Interestingly, some pangenes contains fewer such as [maize_pan_p011111](https://www.greenphyl.org/cgi-bin/sequence.cgi?p=id&sequence_accession=maize_pan_p011111) which contains 3 sequences with one missing in B73 which is the main Maize reference genome sequence.
- maize_pan_p011111 has orthologous sequences with other Poaceae such as rice, wheat, barley sugarcane, Brachypodium and sorghum (see figure below). With the use of only B73, these orthologous relationship would have been missed.


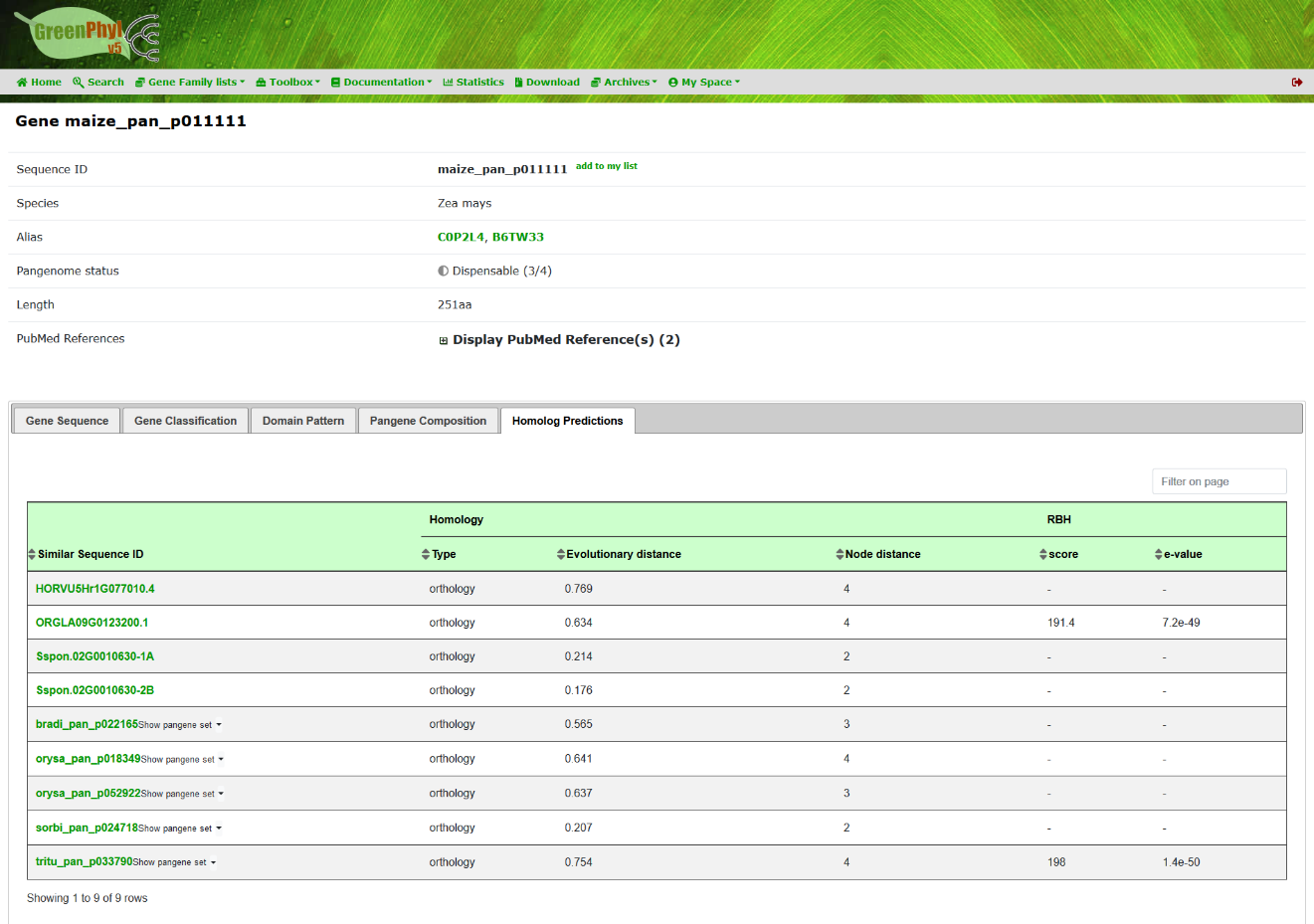


For the whole set of pangenes, the list of orthologous relationships between Maize and Arabidopsis for instance can be listed and exported


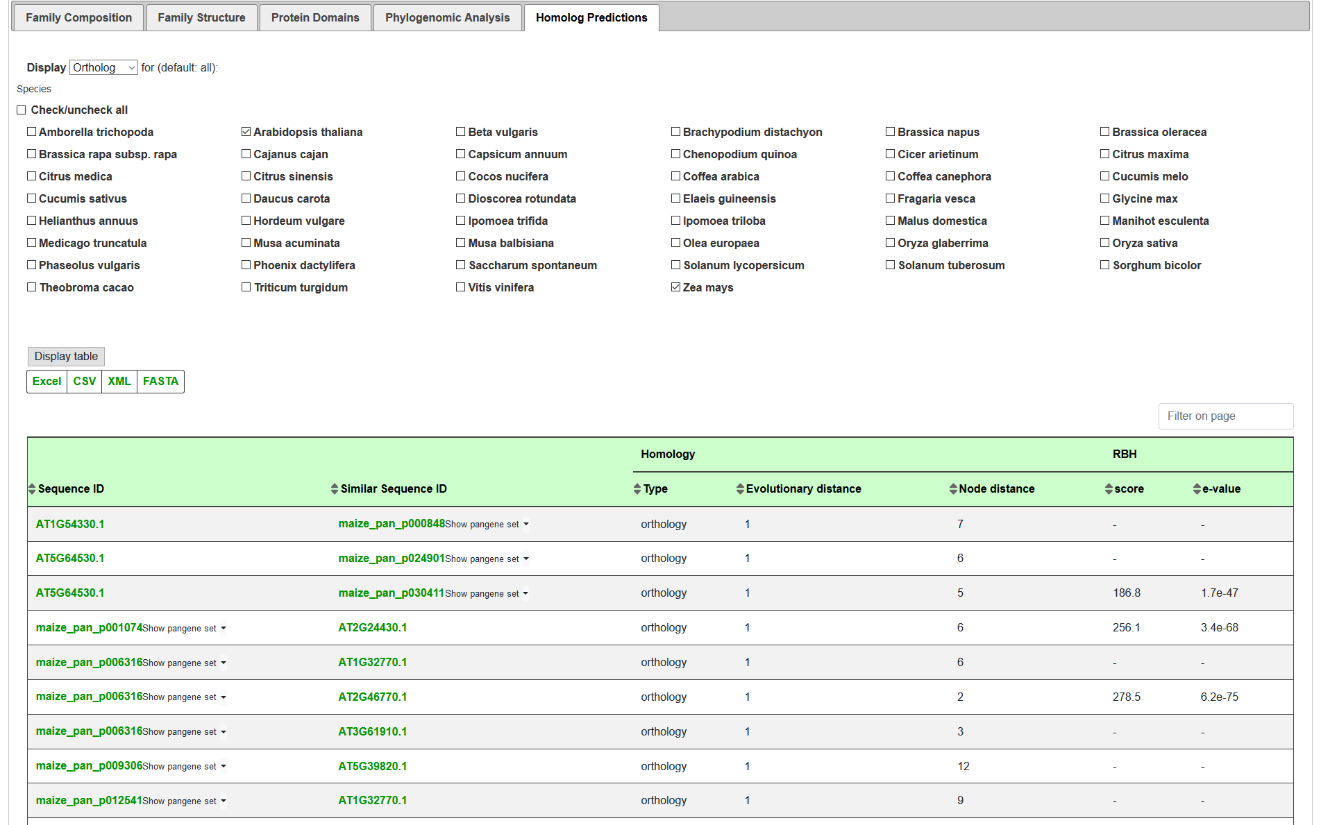


Gene tree can be visualized for the whole gene family or exported in Newick format to use with another software.

- As it contains 5660 sequences, it may not be relevant or suitable to display the whole tree. GreenPhyl proposes an option to prune the gene tree on the fly for a subset of species selected by users. Below an overview of full gene tree, a tree with only Maize and Rice, and a tree with Maize, Rice, Grape and *Arabidopsis thaliana*.


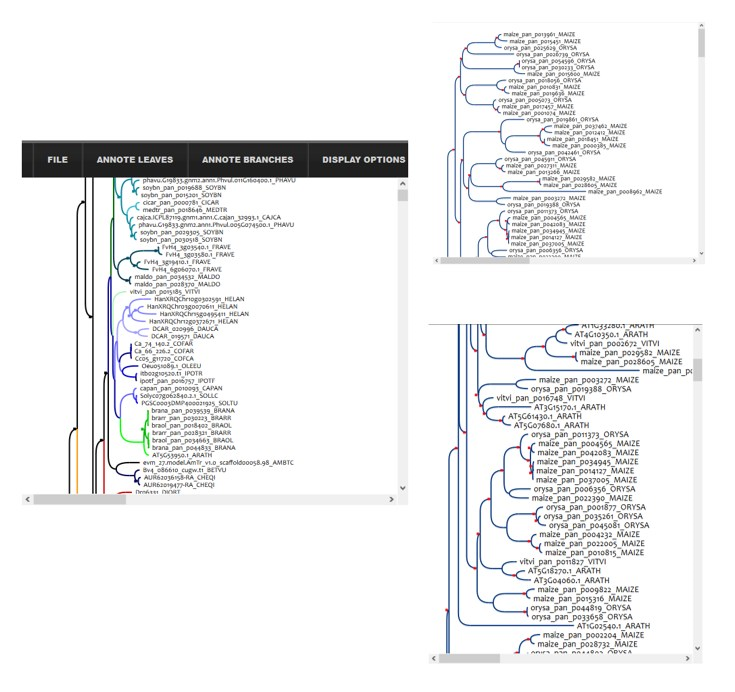


## Use case 2: A user wants to check gene families that followed an evolutionary scenario for gene copy retention between and within species.

In this example, user look for examples where Grape has one gene which would be duplicated in Rice and in Maize. The assumption here is that Maize experienced a relatively recent ancient whole genome duplications (WGD) compared to Rice while Grape did not experience specific ones as illustrated on the image extracted from the Plant Genome Duplication Database (<http://chibba.agtec.uga.edu/duplication/>)


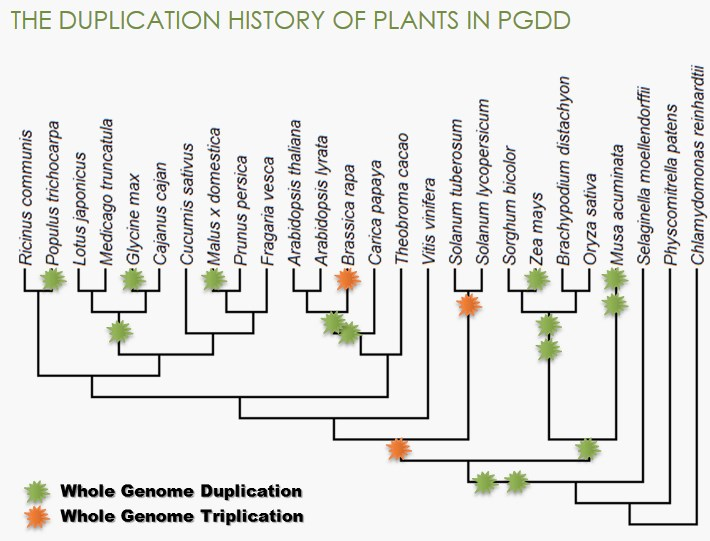


- Draw the evolutionary scenario by create speciation and duplication events, add the species name at the leaf of the tree and finally add constraints on cardinality and sampling using [TreePattern](https://www.greenphyl.org/cgi-bin/treepattern.cgi)


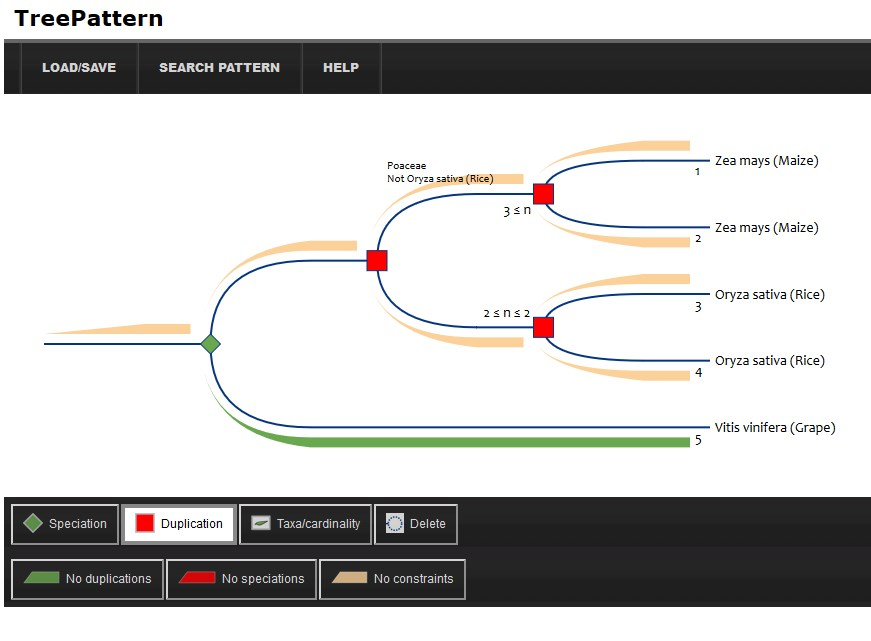


- This example is a pretty complex evolution pattern with stringent parameters returning only 2 gene trees. One of the tree is partially displayed below.


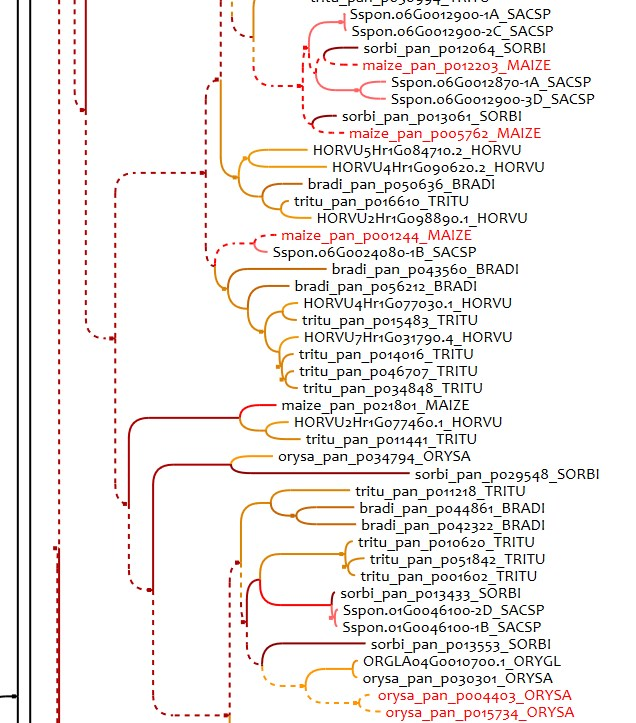


By clicking on one pangene, user can see the gene family classification.

- It corresponds to a family annotated as Cytochrome P450 superfamily containing domain family.
- Without prior knowledge on this family and checking the literature, one can see that duplications on this family with regards to duplication is a relevant question.


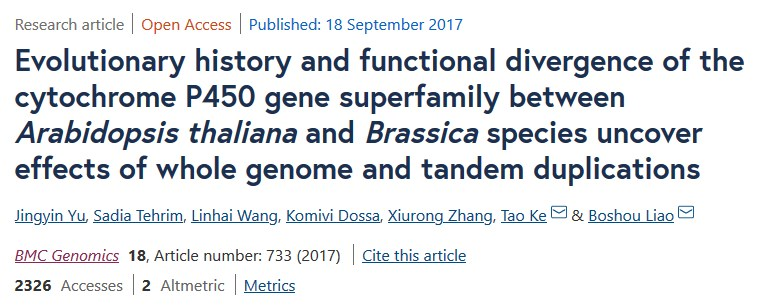


- The study (<https://doi.org/10.1186/s12864-017-4094-7>) conclude that P450 gene superfamily between A. thaliana and Brassica species indicated that the family-specific evolution in the Brassica lineage can be attributed to both whole genome duplication (WGD) and tandem duplication (TD), whereas WGD was recognized as the major mechanism for the recent evolution of the P450 super gene family.
- Looking at the pangene composition, two pangenes belong to the core in maize (found in all 4 genomes) and one pangene to the dispensable (3 out of 4). Others are in dispensable for Rice or Grape. It suggests that the pattern of evolution may not have been conserved for all individuals in the three selected species.

In conclusion, this approach can be a good way to explore gene families without *a priori* and get insights on the dynamics on duplication between and within species. However, conclusions should not be made without a careful examination of the family, possible missing sequences or annotation artefact.

**Use case 3: A user has a candidate gene sequence from an experiment in African Rice and want to find orthologs in Asian rices.**

- The user selects “match sequences” in the toolbox and paste a protein sequence which returns as best hit an *Oryza glaberima* gene


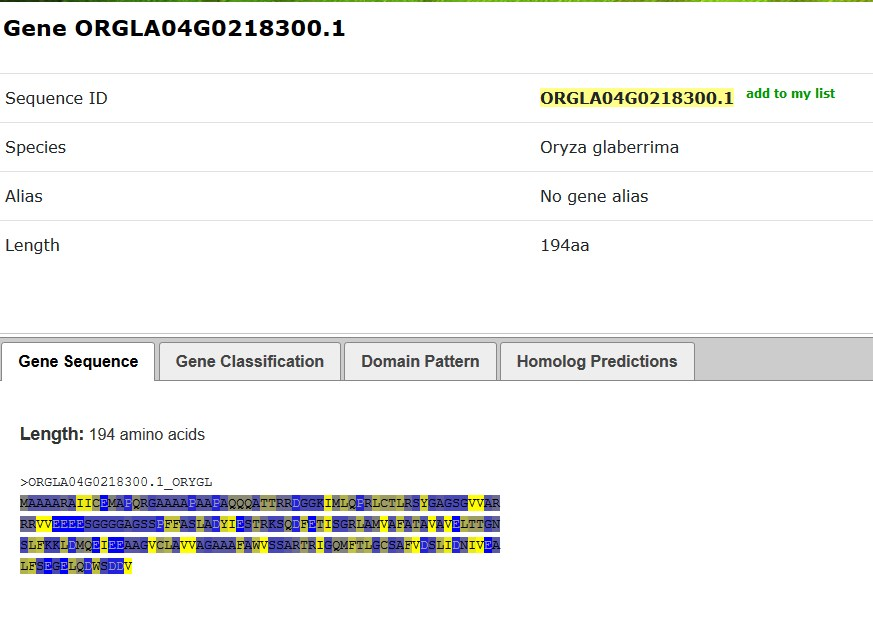


- The sequence belongs to [GP004434](https://www.greenphyl.org/cgi-bin/family.cgi?p=id&mmode=full&family_id=1100442) annotated as **Stress Enhanced Protein 2 (SEP2) homologs family**


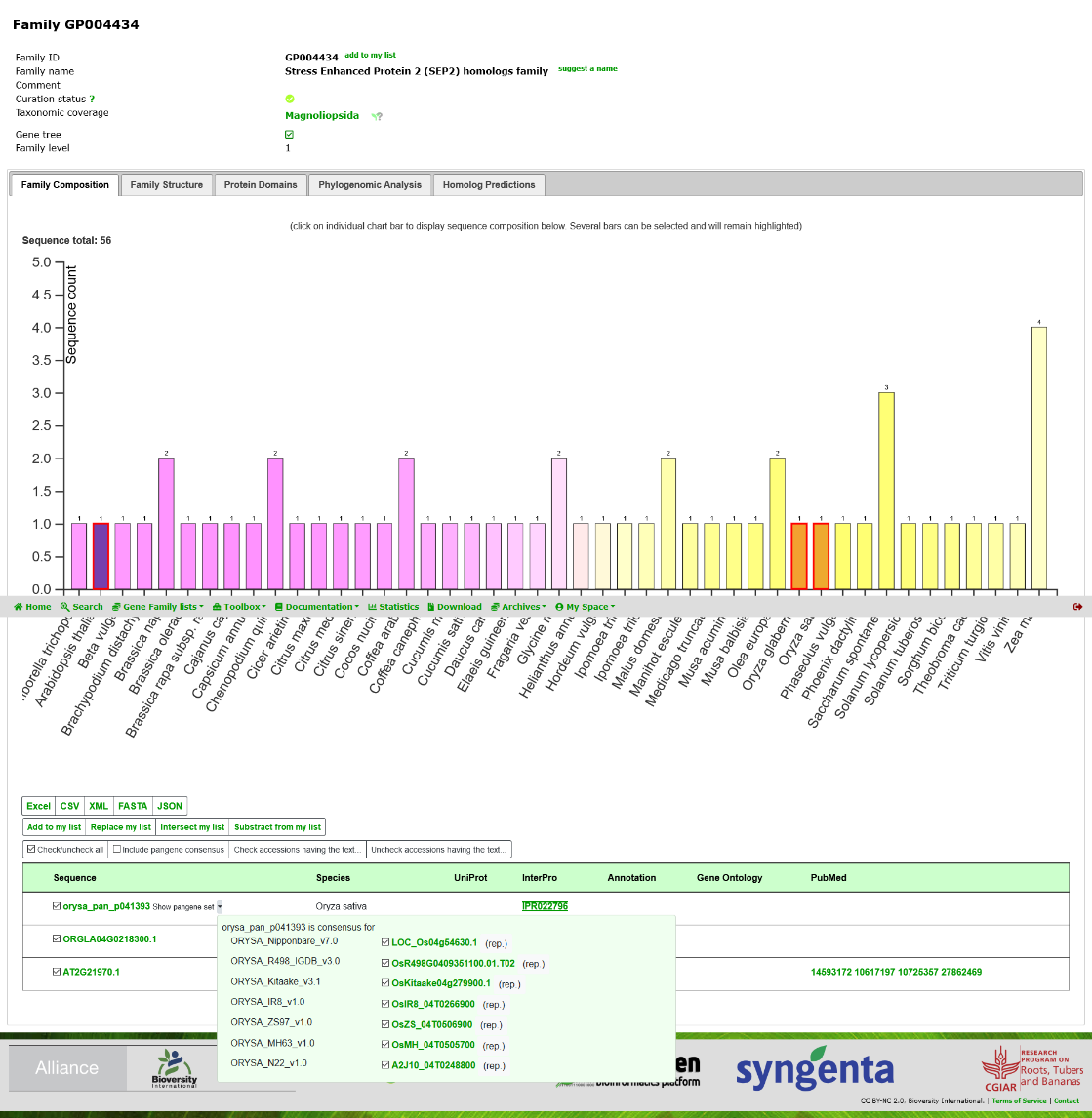


- By clicking on *O. sativa*, it lists one pangene (i.e. [orysa_pan_p041393](https://www.greenphyl.org/cgi-bin/sequence.cgi?p=id&mmode=full&sequence_accession=orysa_pan_p041393)) which represents a sequence in each of the 7 genomes from *O. sativa*.
- The multiple alignment used to generate the pan sequence is available for information.


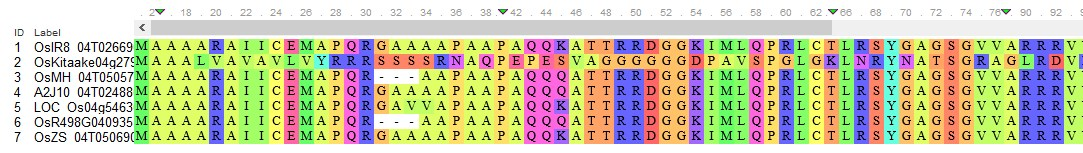


Back to the family page, user can add *Arabidopsis thaliana* (reviewed as [SEP2](https://www.uniprot.org/uniprot/Q9SJ02) in UniProt) for instance and export at once all protein sequences by clicking on the FASTA button for subsequent analyses.


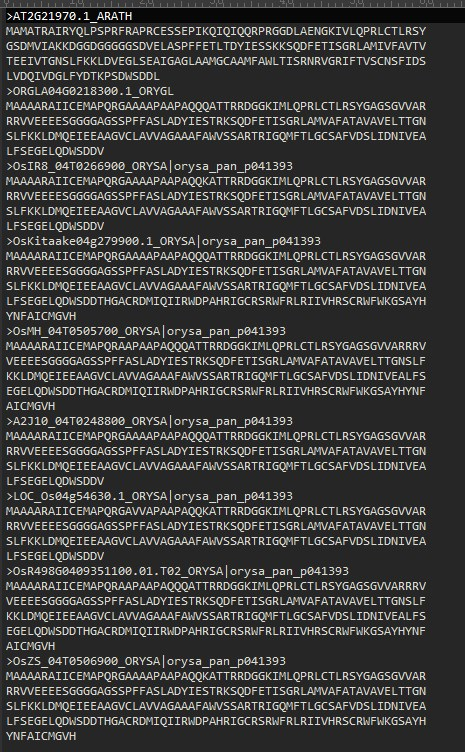


Also, the gene tree can be manipulated to export it with the preferred taxonomic sampling.

An example could be the following gene tree


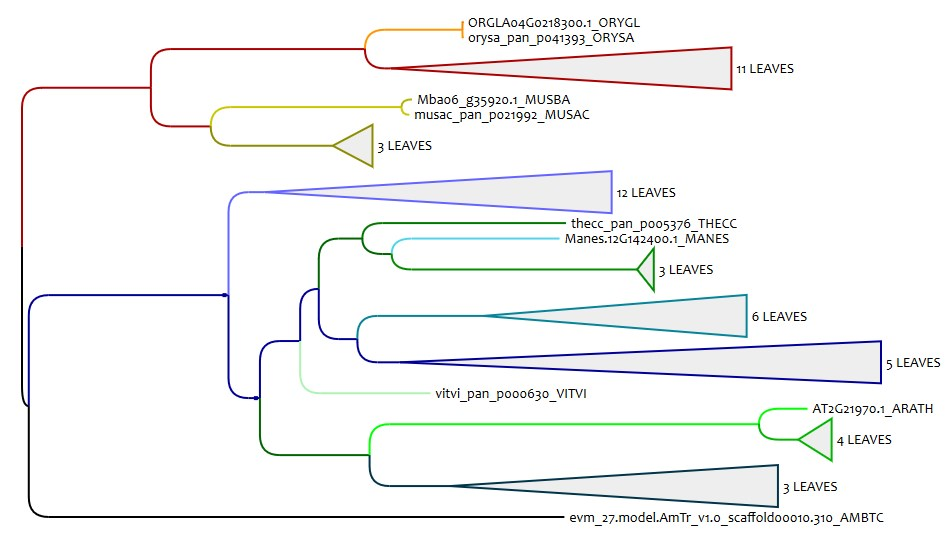

Supplement: gkaa1068_Supplemental_File [file gkaa1068_supplemental_file.docx]
